# Integration of transcriptional inputs at promoters of the arabinose catabolic pathway

**DOI:** 10.1186/1752-0509-4-75

**Published:** 2010-06-02

**Authors:** Carla J Davidson, Atul Narang, Michael G Surette

**Affiliations:** 1 University of Calgary, Department of Biology, BI376b 2500 University Dr. N.W., Calgary, AB. T2N 1N4 Canada; 2 Department of Biochemical Engineering & Biotechnology, Indian Institute of Technology, Hauz Khas, New Delhi 110 016, India; 3 University of Calgary, Department of Microbiology and Infectious Diseases, Room 268 Heritage Medical Research Building, 3330 Hospital Drive NW, Calgary, AB T2N 4N1 Canada

## Abstract

**Background:**

Most modelling efforts of transcriptional networks involve estimations of *in vivo *concentrations of components, binding affinities and reaction rates, derived from *in vitro *biochemical assays. These assays are difficult and *in vitro *measurements may not approximate actual *in vivo *conditions. Alternatively, changes in transcription factor activity can be estimated by using partially specified models which estimate the "hidden functions" of transcription factor concentration changes; however, non-unique solutions are a potential problem. We have applied a synthetic biology approach to develop reporters that are capable of measuring transcription factor activity *in vivo *in real time. These synthetic reporters are comprised of a constitutive promoter with an operator site for the specific transcription factor immediately downstream. Thus, increasing transcription factor activity is measured as repression of expression of the transcription factor reporter. Measuring repression instead of activation avoids the complications of non-linear interactions between the transcription factor and RNA polymerase which differs at each promoter.

**Results:**

Using these reporters, we show that a simple model is capable of determining the rules of integration for multiple transcriptional inputs at the four promoters of the arabinose catabolic pathway. Furthermore, we show that despite the complex and non-linear changes in cAMP-CRP activity *in vivo *during diauxic shift, the synthetic transcription factor reporters are capable of measuring real-time changes in transcription factor activity, and the simple model is capable of predicting the dynamic behaviour of the catabolic promoters.

**Conclusions:**

Using a synthetic biology approach we show that the *in vivo *activity of transcription factors can be quantified without the need for measuring intracellular concentrations, binding affinities and reaction rates. Using measured transcription factor activity we show how different promoters can integrate common transcriptional inputs, resulting in distinct expression patterns. The data collected show that cAMP levels *in vivo *are dynamic and agree with observations showing that cAMP levels show a transient pulse during diauxic shift.

## Background

Early experiments in the utilization of different sugars demonstrated that bacteria will preferentially use glucose over many other carbon sources, a phenomenon termed the glucose effect [1]. Jacques Monod measured the growth curves of *Escherichia coli, Bacillus subtilis*, and *Salmonella enterica *in combinations of different sugars, and found that some combinations resulted in a simple growth curve, while others resulted in a biphasic, or diauxic, curve which is the result of preferential catabolism of glucose in the first growth phase, followed by a lag phase during which the catabolic proteins required for using the second sugar are made [2]. Two mechanisms are responsible for the glucose effect; inducer exclusion and catabolite repression, which are mediated by the phosphoenolpyruvate - dependent transport system (PTS). The first mechanism involves the inhibition of numerous permeases by direct protein-protein interaction with the dephosphosphorylated form of enzyme GluIIA [3], a phenomenon termed inducer exclusion. Also, in the absence of a glucose, phosphorylated enzyme GluIIA will activate adenylate cyclase to increase formation of the second messenger, cyclic AMP (cAMP) [3-5]. cAMP binds to the transcriptional regulator CRP which is involved in regulation of numerous catabolic operons; and it is the maintenance of cAMP at low concentrations during growth on glucose that is the basis of catabolite repression.

The role of the PTS system in the modulation of cAMP levels, and the relative contributions of inducer exclusion and catabolite repression to the glucose effect are controversial. Experiments have shown that while cAMP levels increase during the lag phase, there is no appreciable difference between intracellular cAMP levels during growth on glucose or lactose [6,7], while conflicting studies show a large difference [8,9]. Furthermore there is evidence that there is little relationship between glucose flux and intracellular levels of cAMP, and that *E. coli *increases its intracellular concentration of cAMP before glucose flux decreases [9], which suggests that the PTS system cannot be solely responsible for regulating intracellular concentration of cAMP. However, more recent work has contested this result, and shown that cAMP levels increase as glucose levels reach 10 μM, in concert with increasing phosphorylation of enzyme EIIA^glu ^[8] Despite its role as a global regulator of nutrient status [10] involved in modulating the expression of numerous catabolic operons [4,7,11-15], the contribution of CRP-cAMP to the glucose effect is not completely understood.

The arabinose catabolic pathway is among those positively regulated by CRP-cAMP and is comprised of four operons, all regulated by the same two transcriptional regulators: AraC and CRP. These operons code for genes involved in regulation, transport, and catabolism of arabinose, and each of their respective promoters have binding sites for the transcriptional regulators AraC and CRP that differ in sequence and location relative to the -35 site. The AraC dimer represses transcription of P_araBAD _in the absence of arabinose via loop formation in the regulatory region of the *araC/araBAD *operon [11,16-20]. Binding of arabinose to the C-terminal dimerization domain of the AraC dimer induces flexibility in the dimer, allowing the release of the loop and binding of the dimer to the operator that overlaps the -35 region of P_araBAD_, leading to transcription initiation at this promoter [17,21]. P_araC _is transcribed divergently from P_araBAD _and in this case, AraC acts as a repressor at high concentrations [11,22]. While transcription of P_araC _has a low basal level, the binding of CRP in the *araBAD/araC *regulatory region increases transcription [23]. The promoters of the arabinose uptake operons, P_araE _and P_araFGH_, also have binding sites for both transcription factors. The order and position relative to the -35 region of CRP and AraC operators in P_araE _are the same as P_araBAD _[24]; however, the order of binding sites in P_araFGH _is the reverse, with the CRP site overlapping the -35 region [25]. The differences in sequence and position of transcription factor binding sites determine the strength of interaction between transcription factors and the operator, and transcription factors and the RNA polymerase, which in turn govern the overall transcription rate and kinetics of induction. Thus differing gene expression among the four arabinose regulated promoters is the result of varying integration of the common signals from the two transcription factors and RNA polymerase.

Typically, detailed models of promoter activity [13,26-30] require determining or estimating numerous binding constants, transcription coefficients, degradation coefficients, and intracellular concentrations of each network component, which can be problematic. Alternatively, models can be fit to experimental data in order to estimate parameter sets that explain observed behaviour [14,31], and functions describing transcription factor activity can be estimated from expression data [31,32]. However, non-uniqueness of parameter estimates is a common problem, especially where there are multiple parameters to be estimated. Our knowledge of the integration of transcriptional signals, particularly cAMP-CRP, at sugar catabolic promoters is commonly derived from measurements of steady state promoter activity in gradients of its exogenous inducer, cAMP [14,33]. However, cAMP can be toxic in high concentrations, and evidence has shown that during diauxic shift cAMP levels *in vivo *vary in a non-linear manner, which has led to discussion about the role of cAMP-CRP in diauxic shift [6,7,34-36]. Furthermore, while promoters under positive control by CRP have been used to measure CRP activity within the cell [8,13], the interpretation of these data is difficult because the interaction between RNA polymerase, cAMP-CRP and the promoter are strongly context dependent, result in non-linear transcriptional activation, and will vary from promoter to promoter. Furthermore, cAMP concentrations within the cell have been shown to increase and decrease quite rapidly [6] and a positively controlled promoter may not reflect this. Therefore, it is unclear how we can understand transcriptional dynamics in diauxic shift based on knowledge gained from steady state analyses and without a more direct method of measuring cAMP-CRP dynamics *in vivo*.

Synthetic biology offers an alternative method of measuring the activity of transcription factors *in vivo*. We report here on the development of synthetic reporters for independently measuring *in vivo *activities of CRP-cAMP, AraC-arabinose, and RNA polymerase while investigating diauxic shift in the arabinose regulon. The synthetic transcription factor reporters measure transcription factor activity via repression of a constitutive reporter. We use these synthetic reporters to measure transcription factor activity during diauxic shift to determine whether the rules of integration determined from steady state behaviour can be extended to explain dynamic behaviour of sugar catabolic promoters in changing environments.

## Results

### Sequence Alignment and Construction of Reporters and Synthetic Promoters

**Figure 1:**
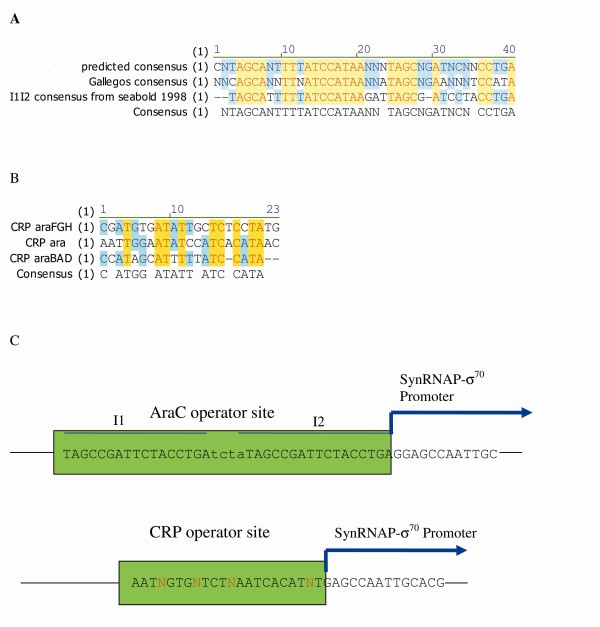
**Sequence and Structure of Synthetic Transcription Factor Reporters**. A. Predicted consensus of I1I2 operators. The predicted consensus from the four AraC regulated promoters is compared to the consensus from Gallegos et al. 1997. Micro. Rev. 61(4): 393 and from Seabold and Schlief 1998. JMB. 278: 529. B. Predicted CRP operator consensus from four promoters. C. Construct of the synthetic transcription factor reporters.

We analyzed sequences of the I1 and I2 AraC operators and CRP operators of the four promoters in order to design primers for the transcription factor reporters. Sequence alignments of the AraC I1 and I2 sites show strongly conserved residues in the I1 portion of the binding site, but more variability in the I2 site. Interestingly, the consensus of Seabold and Schleif [16] has a shorter I2 site by one base pair than the sequenced sites from the arabinose regulon, or than the consensus published by Gallegos and coworkers [37] (Figure 1a), and it was this consensus that provided the strongest binding (Additional file S1, Figure S1). Sequence analysis of the CRP binding site showed that there are five strongly conserved bases (Figure 1b). Based on these calculated consensuses we designed the primers for the transcription factor reporters which included degenerate sites (Figure 1c, Table 1) and cloned them immediately downstream of a synthetic σ^70 ^derivative promoter called synRNAP-σ^70 ^(AATAATTCTTGAAATTTATGCTTCCGGCTCGTATTTTACGTGCAATT). This design resulted in a constitutive promoter whose expression was repressed by the binding of the transcription factor and the inclusion of degenerate sites allowed us to tune the strength of transcription factor binding. A library of reporters was picked and tested for repression in the presence of the inducers (cAMP, arabinose) (Additional file S1 Figure S1, Additional file S1 Figure S2, Additional file S1 Figure S3), and the reporters that showed the strongest repression were named synARA and synCRP and used in all further experiments.

**Table 1:** Primers used in this study

Primer Name	Sequence
araB3'	CATACTCGAGCCATTCAGAGAAGAAACCAA
araB5'	GATGCGGCCGCAGTGACGGCAATGTCTGAT
araC3'	GATGCGGCCGCCGTCAATTGTCTGATTCGT
araC5'	GATGCGGCCGCAGTGACGGCAATGTCTGAT
araE3'	GGGGATCCGGCGTTAAAGCAGATTCCGTAT
araE5'	CGCCTCGAGCCGTGGATGGCGGTTGGCTGG
araF3'	CCCCGGATCCCAGACCAATGGCTGCCAGGG
araF5'short	CCGCTCGAGCGGTTATTACACCATTTC
sigARARS	ATGGATCCTAGTAGCATTTTTATCCATAAGATTAGCGATCCTACCTGAGGAGCCAATTCACG
araCI1I2	ATGGATCCTAGTAGCATTTTTATCCATAAGATTAGCGATCCTACCTGAGGAGCCAATTGCACG
araCI2I2	ATGGATCCTAGTAGCCGATTCTACCTGATCTATAGCCGATTCTACCTGAGGAGCCAATTGCACG
araCDI1I2	ATGGATCCTAGNAGCNTTTTTATCNATATCTATAGCCGATTCTACCTGAGGAGCCAATTGCACG
araCI1I1	ATGGATCCTAGTAGCATTTTTATCCATAAGATTAGCATTTTTATCCATAGGAGCCAATTGCACG
araCDI2DI2	ATGGATCCTAGTNGCCGATTCTACNTGATCTATNGCCGATTCTACNTGAGGAGCCAATTGCACG
sigCRP	GGATCCTAGAATNGTGNTCTNAATCACATNTGGAGCCAATTGCACG

### Gene expression in a gradient of cAMP and Arabinose

**Figure 2:**
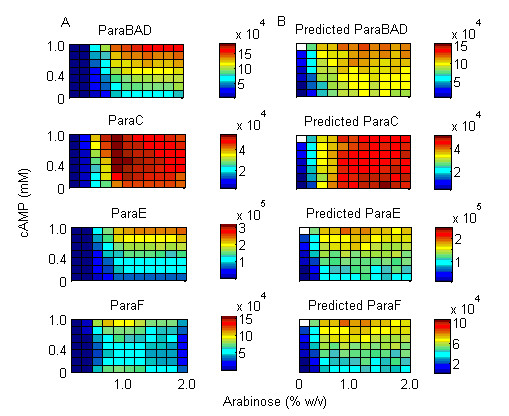
**Gradient of expression in four arabinose regulon promoters**. Graphs show expression data (CPS/OD) 44 minutes post induction. A. Measured expression of the four arabinose catabolic promoters. B. Predicted expression based on the mathematical model.

**Figure 3:**
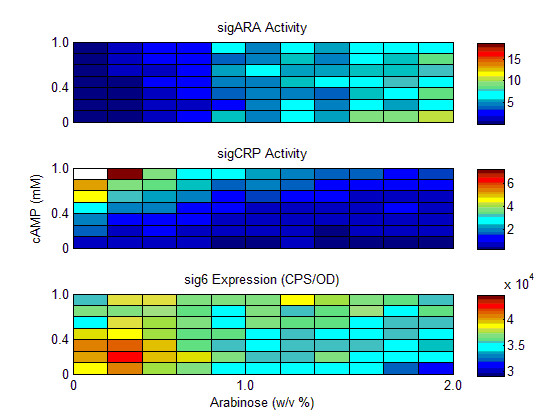
**Activity profiles of the three synthetic transcription factor reporters**. Expression (CPS/OD) was measured in a gradient of cAMP (0-1mM) and arabinose (0-0.2%) at 44 minutes post induction. Activities for synARA and synCRP were calculated using equation #2, and raw expression for synRNAP-σ^70 ^is shown.

To test how concentrations of the two inducers affected gene expression we measured the steady state expression of the four arabinose regulon promoters (P_araBAD_, P_araC_, P_araE_, P_araFGH_) in a combinatorial gradient of both inducers (Figure 2a). Steady state was considered to have been reached by 44 minutes. P_araE _exhibited the strongest expression, and P_araC _exhibited the lowest. All promoters required arabinose in order to detect any reporter expression and responded in a largely step-like manner. cAMP served to modulate expression in the presence of arabinose in a graded manner. P_araC _exhibited slight inhibition in higher concentrations of arabinose and cAMP (Figure 2a). The three synthetic reporters were also assayed under these conditions. synARA and synCRP exhibited graded repression in the presence of their respective small molecule inhibitors (Figure 3). However, there was also an effect of arabinose addition on the repression of synCRP. At all time points synRNAP-σ^70 ^remained largely unaffected by the concentration of either small molecule effector (Figure 3).

### Promoter Induction Kinetics During Diauxic Shift

The kinetic behaviour of the four arabinose regulon promoters was investigated in conditions of diauxic shift which measures promoter and transcription factor activity in conditions of rapidly changing endogenous cAMP concentrations, from low (high glucose) to high (all glucose consumed) cAMP. Briefly, the optical density and *luxCDABE *expression of both the catabolic promoters and transcription factor reporters was measured every 4 minutes during exponential phase, in a medium with subsaturating glucose and saturating arabinose. From these data, promoter expression (CPS/OD) and transcription factor activity for AraC-arabinose and CRP-cAMP was calculated according to equation 2.

**Figure 4:**
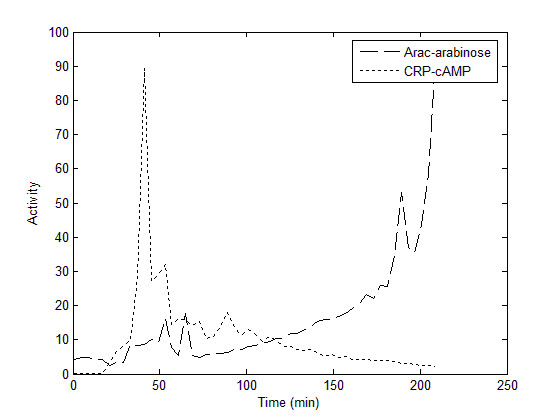
**Calculated transcription factor activity during diauxic shift**. The period of glucose starvation is indicated by the peak in CRP-cAMP activity. Activity is calculated using equation 2 from measurements of synRNAP-σ^70^, synCRP and synARA expression. These profiles represent mean expression from n = 7 experiments with time 0 normalized to the start of glucose exhaustion.

In the diauxic shift experiments, the exhaustion of glucose varied between experiments. Therefore, we adjusted the start of the experiment to be twenty minutes before the increase in synCRP activity. CRP-cAMP exhibited a strong peak of activity in all assays, which then rapidly returned to baseline activity. Conversely, AraC-arabinose activity increased in an exponential manner after the reduction in cAMP-CRP activity (Figure 4).

### Model Development and Analysis

**Figure 5:**
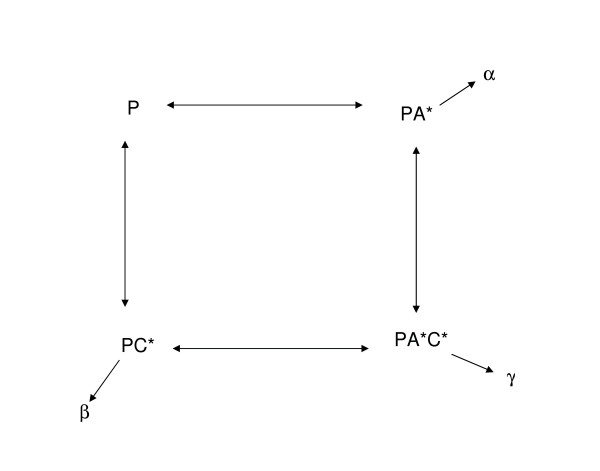
**Simple mathematical model of promoter expression in arabinose regulon**. The promoter, P, can be bound to AraC-arabinose (A*), CRP-cAMP (C*), or both. Transcription is theoretically possible in any of these states and is described by transcription activation rates α, β and γ respectively. Note that these activation rates include binding equilibria for transcription factor binding, and there is no cooperativity.

The model of promoter expression as a function of measured transcription factor activity (Figure 5) was fit to data of steady state expression using non-linear least squares fitting (procedure nls, R [38]). The results of the fit are presented in Table 2. All models had statistically significant estimates for α and γ; however, the estimates for β for P_araBAD_, P_araC_, P_araE _and P_araFGH _are all insignificant at *p *= 0.05. In these cases the model was refit by removing this term and the residual squared error (RSE) and Akaike information criterion (AIC) were calculated. In every case, both RSE and AIC showed improvement in model fit by removing this term. Model fit was tested by predicting gradient expression given measured synthetic transcription factor activity (Figure 2b). These simulations showed that the fit predicted steady-state gradient expression in all four transcription factor reporters quite well.

**Table 2:** Fitted coefficients from fit of model to steady state expression.

	α	β	γ
P_araBAD_	2.535***	NS	4.347***
P_araC_	1.656***	NS	1.078***
P_araE_	1.684***	NS	7.726***
P_araFGH_	0.6546**	NS	3.344***

Significance codes: *** p = 0, ** p < 0.001, NS = not significant

**Figure 6:**
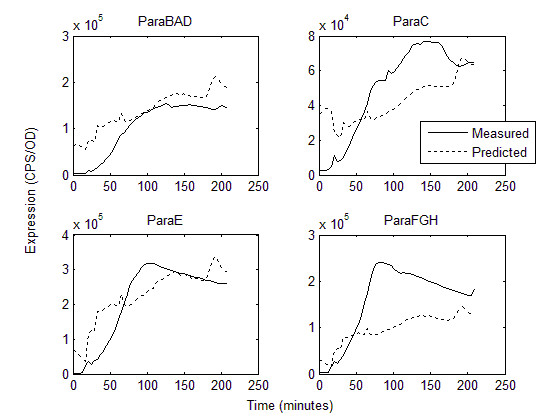
**Observed and predicted kinetics of expression (CPS/OD) of four AraC regulon promoters, P_araBAD_, P_araC_, P_araE_, P_araFGH_, during diauxic shift**. Predictions are calculated using TF activity profiles (Figure 4) and transcription activation rates estimated from model fits to steady state expression data (Table 2).

The models derived from this fit were then tested for their ability to predict induction in diauxic shift (Figure 6). The predicted expression was calculated by using the estimates for α and γ derived from each fits to steady state data (Table 2), and the calculated transcription factor activity measured by the transcription factor reporters (Figure 4). The models predicted expression that mimicked measured expression in both magnitude and timing, with the exception of P_araFGH_. In this promoter, the measured magnitude of expression was approximately two-fold higher than predicted. Also, the dynamics of expression in P_araC _were slightly underestimated by our model.

## Discussion

Three synthetic transcription factor reporters, synARA, synCRP, and synRNAPσ^70^, were used to measure the *in vivo *steady state activity of AraC, CRP, and RNA polymerase in gradients of arabinose and cAMP. This information was used in a mathematical model to determine the rules of integration of common transcriptional inputs at the promoter level. The test of the model was to determine whether these rules could predict promoter behaviour during diauxic shift when AraC-arabinose and cAMP-CRP activity were measured *in vivo*. At steady state, the transcription factor reporters showed a graded response to the concentration of their respective small molecule effector, demonstrating that repression is an effective method for measuring transcription factor activity that obviates the need for estimating various binding and degradation constants. There was a slight amount of cross talk between small molecule effectors; for example, the synCRP reporter showed slight inhibition as the concentration of arabinose increased (Figure 3). This may be due to the internal regulation of cAMP as the arabinose is catabolized. The synRNAPσ^70 ^reporter remained largely unaffected by arabinose or cAMP concentration (Figure 3). A simple mathematical model based on measured transcription factor activities was sufficient to reproduce steady state behaviour (Figure 2), and furthermore, could predict dynamic behaviour during diauxic shift using measured transcription factor activities (Figure 6). These results suggest that the repression of a constitutive promoter is an efficient and accurate method to measure transcription factor activity *in vivo*, without the need to measure binding coefficients and intracellular concentrations biochemically.

Induction during diauxic shift is the most biologically realistic environment to test the response of the four promoters to transcription factor activity. Furthermore, the role of cAMP in transcriptional activation during diauxic shift is in question due to the complex behaviour of cAMP as glucose is consumed [6,7,34-36]. The model fit to steady state expression data is capable of predicting the major aspects of induction in diauxic shift, and suggests that both CRP and AraC are capable of binding their operators and interacting with RNA polymerase as long as repression by AraC is relieved. The significance of the γ term (Table 2) suggests that both arabinose and cAMP are required for full transcription (AND logic), similar to earlier results [39,40]. However, the additional significant α term (Table 2) indicates that AraC alone can modulate transcription independently of cAMP. This suggests that the logic function performed by all the arabinose catabolic promoters is more complex, similar to that measured for P_lacZ _[14].

The measurement of cAMP-CRP activity during diauxic shift in this study (Figure 4) confirms other observations of cAMP concentration in these conditions [6]. Before the decline of growth rate, decrease in glucose uptake, or reduction in glucose flux, cAMP concentration within the cell changes rapidly, rising quickly, then soon returning to basal activity once the secondary sugar has been detected [9]. This burst of activity has been demonstrated by other investigators [6]. Because cAMP changes so rapidly, it was unclear to us whether models derived from steady state activity could be used to understand dynamical behaviour. Surprisingly, the model derived from steady state behaviour does predict the salient aspects of diauxic shift, though model fits for P_araFGH _and P_araC _are not as accurate as those for P_araE _and P_araBAD _(Figure 6). This may reflect specific aspects of transcription in these promoters that is not properly modeled. For example, the relative positions of the CRP and AraC operators in P_araFGH _are opposite that of ParaE, with the CRP operator overlapping the -35 site [25]. This suggests that the effect of CRP-cAMP at this promoter may be stronger than AraC, and may include some cooperativity that is not included in the model. Also, at P_araC_, AraC can act as a repressor at the O_1 _site [11,22]. Repression was not included in this analysis and indeed it is clear that the drop in expression at high arabinose concentrations at this promoter is not adequately modeled. The role of AraC as a simple activator may be oversimplified, as it remains unclear what role AraC plays in the kinetics of induction, given that it is most active at time points after the promoters have been activated (Figure 4). This may indicate that the primary role of arabinose binding to AraC is to relieve repression. That the predicted expression is higher at early time points than measured suggests that there are elements of repression via AraC that the model does not reproduce. AraC has been demonstrated to be required to initiate transcription by helping to recruit RNA polymerase to the -35 site [17,18,20,41,42]; however, the protein is always present at I2 which is immediately adjacent to the -35 site. Therefore, changes in concentration of AraC, which would be measured by our reporter, may happen long after the resident AraC has fulfilled its role. Conversely, the low early activity of synARA may simply be an artefact of this reporter being relatively low strength.

Mathematical models of transcriptional regulation have either relied on estimates of *in vivo *binding affinities, concentrations and reaction rates [30], or have estimated these "hidden functions" [31,32] from transcriptional data. The first technique has the complication of having to measure numerous quantities *in vitro*, which is both difficult and hard to relate to *in vivo *conditions. The second technique has the possibility of estimating non-unique parameter sets and remains a substitute for being able to measure *in vivo *transcription factor activity in real time. While cAMP concentrations *in vivo *have been estimated from non-native CRP-dependent promoters [8,13], the interaction between RNA polymerase and the transcription factor at the promoter results in a non-linear activation of transcription. The non-linearity in transcription activation complicates the interpretation of the rate at which *in vivo *cAMP concentrations are increasing or decreasing. For these reasons, determining *in vivo *concentrations of small molecule activators, or the activity of their cognate transcription factors, has been exceedingly difficult. Therefore, we used synthetic biology techniques to develop transcription factor reporters that can give an indication of transcription factor activity in real time. The synthetic transcription factor reporters consist of a constitutive promoter followed by the operator binding site for the transcription factor of interest. It is relatively simple to develop synthetic reporters for intercellular concentrations of effectors by cloning a library of reporters with degenerate operator binding sites [43] (Figure 1c, Additional file S1, Figure S1, Additional file S1 Figure S2, Additional file S1 Figure S3). Experiments showed that not only were the reporters capable of giving sensitive, linear and real time indications of *in vivo *transcription factor activity (Figures 3, 4), but that simple models were capable of relating these measurements to promoter activity (Figure 2) even in a dynamic experiment such as during diauxic shift (Figure 6). In other words, using these promoters we measured real time *in vivo *transcription factor activity and determined their rules of integration at the promoter level. This is a marked improvement on methods of measuring small molecule concentrations in real time.

## Conclusion

Preferential catabolism of sugars and signalling of the nutritional state is regulated by complex interactions of sugar intake systems, adenylate cyclase, and transcriptional regulation. This work demonstrates the development of novel synthetic reporters that measure the transcription factor activity *in vivo *and obviate the need for the estimation of numerous parameters. Because cAMP activity during diauxic shift shows a rapid pulse of activity, its role in regulation of catabolic promoters during diauxie has been contentious [7,34-36]. However, the integration of information from common transcription factors is mediated by the relative strength of α, β and γ, and this analysis shows that understanding these rules is sufficient to predict the salient features of gene regulation during diauxic shift, without *in vitro *estimation of numerous biochemical coefficients.

## Methods

### Strains and conditions

The *Escherichia coli *strain used in this study was MG1655. All strains were cultured aerobically at 37 °C in Luria Bertani (LB) broth (Invitrogen Canada, Burlington Ontario) or M9 minimal medium (Becton Dickinson Canada Inc., Mississauga, Ontario, Canada) supplemented with 0.1% casamino acids (Becton Dickinson Canada Inc.) and either 0.5% glycerol or 0.1% glucose. Kanamycin was included in liquid and solid media at a concentration of 50 μg/mL as required.

### Sequence Alignment and Construction of Reporters and Synthetic Promoters

The complete intergenic region for the promoters P_araC_, P_araBAD_, P_araE _and a truncated fragment of the *araFGH *intergenic region including all known regulatory elements were PCR amplified using primers listed in Table 1. The PCR products were purified, digested with *Xho*1 and *Bam*H1 (New England Biolabs, Ipswitch MA.) and ligated into the low copy *luxCDABE *reporter plasmid pCS26-Pac [44]. Positive recombinant plasmids were identified by luciferase expression and confirmed by DNA sequencing.

Previous work describes the development of a library of synthetic σ^70 ^promoters of varying strengths (Pabbaraju and Surette, unpublished results). The transcription factor reporters were built from a constitutive σ^70 ^promoter from this library that exhibited a medial level of expression called synRNAP-σ^70 ^(AATAATTCTTGAAATTTATGCT TCCGGCTCGTATTTTACGTGCAATT). Alignments of the AraC binding site and CRP binding site in the AraC regulon were done in the program AlignX (Invitrogen, Canada) and these consensus binding sites were the basis of the transcription factor reporter libraries. The consensus binding site, with four degenerate bases, was added to the 3' primer for the synRNAP-σ^70 ^promoter. This design placed the operator region immediately downstream of the -10 position of the constitutive synRNAP-σ^70 ^promoter, and the addition of degenerate bases allowed us to screen a large number of clones for different levels of activity. Primers designed for synARA clones included binding regions that were based on a single I1 site, or combinations of I1I1 and I1I2 sites with and without degenerate bases (Table 1). We also tested the slightly different I1I2 consensus sequence of Seabold and Schlief [16]. The PCR products were purified and cloned into the pCS26-Pac plasmid. The ligation reactions were transformed into Electromax electrocompetent DH10b cells (Invitrogen Canada), the plasmid library recovered from the liquid cultures, and retransformed into chemically competent MG1655. The library was picked into 96 well plates and screened for responsiveness to the addition of the relative effector. The transcription factor reporters showing the strongest repression were selected and named synARA and synCRP.

### Steady State Promoter Expression

Overnight cultures of each reporter were diluted 1/600 into fresh LB medium and grown to half exponential phase (three hours, OD_600 _0.1-0.2). 50 μl of each reporter was then added to each well in a 96 well plate containing 50 μl LB and gradients of arabinose (0-0.2%) and cAMP (0-1 mM). The plates were read in a multiwell plate reader (Wallac Victor 1420 multilabel counter) at 4 minute intervals, with agitation (30 seconds, 2.0 mm orbital shake prior to measurement) for an hour and a half. This allowed for fine-resolution temporal mapping of promoter activity over 96 different combinations of arabinose and cAMP concentrations (after [14]). Data was normalized to optical density prior to analysis.

### Specificity of transcription factor reporters

To test for specificity of the transcription factor reporters, luciferase expression was measured in a gradient of both arabinose and cAMP. Overnight cultures of synARA, synCRP and synRNAP-σ^70^was diluted 1/600 into fresh LB medium and grown to half exponential phase (three hours, OD_600 _0.1-0.2). 50 μl of each reporter was then added to each well in a 96 well plate containing 50 μl LB and gradients of arabinose (0-0.2%) and cAMP (0-1 mM). OD and luminescence were measured using a multiwell plate reader (Wallac Victor 1420 multilabel counter) at 4 minute intervals, with agitation (30 seconds, 2.0 mm orbital shake prior to measurement) for an hour and a half. This allowed for fine-resolution temporal mapping of transcription factor activity over 96 different combinations of arabinose and cAMP concentrations [14].

### Induction Kinetics from Diauxic Shift

For diauxic shift assays, the cultures were grown overnight in M9 medium plus glucose, diluted 1/600 in the same medium and grown to half exponential phase (three hours, OD_600 _0.1-0.2). The cultures were then rinsed twice in M9 medium without a carbon source, then added to the 96 well plate with saturating arabinose (0.1%) and subsaturating glucose (0.0001%, 0.0002%, 0.001%, 0.005%, 0.01%). The plate was measured in the same manner as above. Luciferase expression in four minute intervals was measured as before for three hours. All gene expression assays were performed in triplicate, and data was normalized to optical density prior to analysis.

### Model Development and Analysis

The concentration of bound arabinose-AraC and cAMP-CRP multiplied by their relative binding constants at the operator are termed activities and designated A* and C* respectively. These activities can be measured *in vivo *using synthetic promoters, which are comprised of a constitutive promoter, synRNAPσ^70^, preceded by the operator of interest (Figure 1c). Using the synARA reporter as an example, the binding of arabinose-AraC at its relative operator site results in repression of the constitutive reporter, thus expression can be modelled using a simple model of transcriptional repression [39,45]:(1)

where SA is the measured expression of synARA, α_σA _is the transcription activation rate, the term A* describes the activity of arabinose-AraC, and *T_σA _*describes basal transcription. The activity, or proportion of AraC-arabinose bound at the promoter, can be estimated by rearranging 1:(2)

Because transcription of this reporter (SA) and the basal transcription rate (T) are measured quantities, and the activation rate is by definition the difference between fully repressed and fully unrepressed expression [39] and thus can be estimated from measured data (*SA*_t _- *T*_synRNAPσ7*0*t_), we can easily calculate the quantity A* for every combination of inducers, or for every time step in a diauxic shift experiment. In instances where equation 2 resulted in a negative value, the results were normalized to 0. Identical arguments apply to calculating the activity of cAMP-CRP from the measured expression of synCRP.

Following Figure 2, we developed a simple model of transcription activation of the four arabinose catabolic promoters, P_araBAD_, P_araC_, P_araE_, and P_araFGH_. Because transcription factor activity is being measured *in vivo*, we can neglect sub reactions such as binding of arabinose to AraC, or cAMP to CRP, and binding of these two complexes to their respective operator sites. When both transcription factors are interacting at the same promoter there is the possibility of transcription due to the binding of one transcription factor, transcription due to the binding of the other, and transcription due to the binding of both (Figure 5). Let α = transcription due to activity of arabinose-AraC (A*), β = transcription due to activity of cAMP-CRP (C*), and γ = transcription due to the activity of both (A* × C*). These transcription activation rates comprise the "rules of integration at the promoter," and describe how the promoter integrates common transcriptional inputs. It is also conceivable that RNA polymerase may alter genome-wide activity depending on cellular conditions. This would have the property of scaling the whole term describing transcription due to binding of AraC and CRP according to the factor σ. This quantity is measured by the reporter synRNAσ^70 ^and therefore can be included in the equation. Assuming that each of these is additive and contributes to the overall transcription of P_araBAD _we get the following equation:(3)

where B represents expression of P_araBAD_, σ is the measured expression of synRNAPσ^70^, *T_B _*represents basal transcription, and α, β, γ are as defined above. Analogous models were used to examine the expression of the other three catabolic promoters. Dynamic expression during diauxic shift is described by the following equation:(4)

where μ is loss due to degradation and dilution as the cell grows. For long lived proteins (protein life significantly longer than cell division time) the term can be simplified to dilution. The half lives of AraB, AraE, and AraFGH are not available, to the best of our knowledge. However, the half life of β-galactosidase has been measured at 8.33 × 10^-4 ^min^-1 ^[30] which, if one assumes a half hour doubling time, corresponds to 0.02499/cell cycle. If we assume that the half lives of the catabolic proteins are similar, then loss due to dilution during exponential growth is more significant than loss due to degradation. Thus after Rosenfeld (2002) the term describing loss resolves to(5a)

where τ corresponds to cell cycle time, determined during time course expression assays, However, the half life of AraC has been determined to be 60 minutes [46], resulting in a degradation constant of 0.0833 min^-1^. In this case, loss due to degradation cannot be discounted, thus both degradation and dilution are included in the model of P_araC _regulation, and degradation in this particular case resolves to(5b)

### Model Fitting

If we assume that(6)

then data evaluated from equation 2 can be substituted in equations 3 and 4. The two terms in equation 6 are not equivalent, thus the values estimated for α, β and γ do not exactly correspond to activation coefficients but rather include the ratio of the binding of the transcription factors to the synthetic promoters (K_1syn_) to the binding of the transcription factors to the promoters in their natural context (K_1_). However, the transcriptional activity measured by the synthetic promoters is proportional to actual activity *in vivo*, so that coefficients estimated by model fitting illustrate relative strengths of inputs at each promoter. The coefficients α, β and γ in equation 3 are determined by model parameterization. If any of these coefficients resolves to zero or is insignificant, then the corresponding term can be dropped from the equation and a simpler model refit to the data. Models were fit with nonlinear least square fitting using the function nls in the statistical package R [38]. Model fit was evaluated by comparing residual sum of squares and Akaike's Information Criterion.

The functional form and scaling coefficients predicted from fitting expression data to measured steady state transcription activity should be sufficient to predict kinetics of induction in a dynamical model. Thus the fitted coefficients were then used to predict induction both in steady state and during diauxic shift using measured transcription factor activities and equation 4. Data from multiple diauxic shift experiments and gradient expression assays (n = 7 induction from diauxic shift, n = 3 gradient expression) were averaged.

## Authors' contributions

CJD Performed all cloning, experiments and data analysis, developed and fit the model, and wrote the manuscript. AN Assisted with development of the model, edited and approved the manuscript. MGS Developed original concept, guided experiments, edited and approved the manuscript.

## Supplementary Material

Additional file 1 **Supplemental Figures**. Three additional figures showing sensitivity of library of synthetic transcription factor reporters. Click here for file
