# Supplementary material for: Integration of transcriptional inputs at promoters of the arabinose catabolic pathway
Source: BMC Syst Biol. 2010 Jun 2;4:75. doi: 10.1186/1752-0509-4-75 (PMC2893085; doi:10.1186/1752-0509-4-75)
Supplement: Additional file 1 — Supplemental Figures. Three additional figures showing sensitivity of library of synthetic transcription factor reporters. [file 1752-0509-4-75-S1.DOC]

Figure S1. Mean and 95% confidence intervals of ratio of sigARA expression in 0.1% arabinose to expression in LB. Expression is normalized to sig6 expression in either condition.

Figure S2. Mean and 95% confidence intervals of ratio of sigCRP expression in repressive and derepressive conditions. 1mM cAMP to expression in LB (light grey) and ratio of sigCRP expression in 0.2% glucose to expression in LB (dark grey). Expression is normalized to sig6 expression in either condition.

A

B

Figure S3. Ratio of expression in repressive and control conditions of different sigARA and sigCRP isolates in a gradient of arabinose or cAMP. A: Ratio maximum expression normalized to sig6 of sigARA isolates in LB and 0.2, 0.15, 0.1, 0.05, 0.01. 0.005, 0.001 and 0% w/v arabinose. SigARARSI1I2 is shown on the second y-axis. B: Ratio maximum expression normalized to sig6 in LB and 0, 1.25, 1.875, 2.5, 3.125, 3.75, 4.4, 5.0mM cAMP. sigCRPG1 is shown on the second y axis.
